# Scarf osteotomy for hallux valgus surgery: determining indications for an additional Akin osteotomy

**DOI:** 10.1186/s13018-023-03908-0

**Published:** 2023-06-16

**Authors:** Yogen Thever, Jerry Chen Yongqiang, Toh Rong Chuin, Nicholas Yeo Eng Meng

**Affiliations:** Department of Orthopaedic Surgery, Singapore General Hospital, 20 College Road, Academia, Level 4, Singapore, 169865 Singapore

## Abstract

**Introduction:**

There is a lack of clear indications to carrying out an Akin osteotomy in addition to scarf osteotomy. Recent studies have shown that a proximal distal phalangeal articular angle (PDPAA) of > 8° as an indication to carrying out additional Akin osteotomy correlates with better radiological outcomes with lesser risk of recurrence. Our study aimed to validate carrying out the additional Akin osteotomy at a PDPAA > 8° while looking into functional outcomes which have not been studied.

**Methods:**

Patients who underwent scarf and combined scarf and Akin osteotomy in our institutional registry was identified. Patient reported outcome measures were compared between patients who underwent scarf and combined scarf and Akin osteotomy. The Visual Analogue Scale (VAS), American Orthopedic Foot and Ankle Score (AOFAS), Short Form-36 Physical Component Score (PCS) and Mental Component Score (MCS) were measured pre-operatively and across a follow up period of 2 years.

**Results:**

A total of 212 cases were identified. At a PDPAA > 8, there was no difference in VAS, AOFAS, PCS and MCS between patients that had isolated scarf osteotomy and those that received combined scarf and Akin osteotomy pre-operatively, and at 6 months. However, at 2 years post-operatively, patients that received scarf and Akin osteotomy had a significantly better AOFAS score as compared to patients with isolated scarf osteotomy (82.3 ± 15.3 vs 88.4 ± 13.0, *p* = 0.0224). On the contrary, at a PDPAA < 8, patients who underwent combined scarf and Akin osteotomy had a significantly lower VAS score at 6 months (1.16 ± 2.16 vs 0.321 ± 1.09, *p* = 0.00633) and 2 years (0.698 ± 1.73 vs 0.333 ± 1.46, *p* = 0.0466). They also had a higher AOFAS score at 6 months (80.7 ± 14.3 vs 85.4 ± 12.5, *p* = 0.0123) and 2 years (83.0 ± 14.0 vs 90.7 ± 9.9, *p* < 0.0001).

**Conclusion:**

PDPAA > 8° can serve as a valid indication to carrying out additional Akin on top of scarf osteotomy based on functional outcomes. However, further studies should investigate a PDPAA threshold that is lower than 8°, which can potentially allow more patients to receive the additional Akin osteotomy that can bring better functional outcomes.

## Introduction

Hallux valgus is a common condition [1, 2] where patients experience significant disability and pain, especially localized to the bunion area with footwear issues, commonly impairing quality of life [2–4]. Typical treatment involves a trial of conservative management with analgesia and footwear modification. Failing which, surgical management is indicated if patients experience persistent pain and inflammation in the affected bunion, with transfer metatarsalgia. Various techniques for surgical correction of hallux valgus deformity have been described in the literature, including the frequently used scarf osteotomy [5–8]. It involves correction at the metatarsal level [5] and has been shown to be associated with good functional and radiological outcomes [5, 7, 9, 10].

Theoretically, carrying out an additional Akin osteotomy in addition to scarf osteotomy should provide better correction as Akin osteotomy would target hallux valgus on a phalangeal level, correcting for hallux valgus interphalangeus as well, which is commonly found intra-operatively [11]. Hallux valgus deformity contributed by phalangeal deformity is commonly reflected by an abnormal hallux valgus interphalangeus angle. However, this has been found to be unreliable due to significant variation across patients due to phalangeal pronation or the irregular geometry of the distal phalanx [12]. More recently, proximal distal phalangeal articular angle (PDPAA) has been found to reflect the degree of hallux valgus interphalangeus more consistently [13].

The Akin osteotomy technique was first described by Akin [14], with various studies showing good outcomes since [15–17]. There is, however, a gap in current literature describing the indications for carrying out Akin osteotomy in addition to scarf osteotomy. Furthermore, few studies have compared functional outcomes in patients who have undergone a combined scarf and Akin osteotomy against patients who have only undergone scarf osteotomy. A recent publication by Kaufmann et al. aimed at describing indications to carrying out a concomitant Akin osteotomy by comparing radiological outcomes and recurrence, found that a PDPAA angle of 8° served as a reliable cut off where Akin osteotomy is indicated [9, 18]. It is important in defining when an additional Akin osteotomy should be carried out on top of scarf osteotomy due to potential further complications that an additional procedure may cause. In addition, apart from defining indications for additional Akin osteotomy based on radiological outcomes, that of functional outcomes were not addressed.

The purpose of our study was hence to compare functional outcomes after scarf osteotomy, as compared to combined scarf and Akin osteotomy at a PDPAA cutoff of 8 degrees, to validate and provide additional value to the study carried out by Kaufmann et al. [9, 18]. We hypothesize that a PDPAA cutoff of 8 degrees is an appropriate indication for combined scarf and Akin osteotomy based on post-operative functional outcomes.

## Methods

### Patient selection

Our study was approved by a centralized institutional review board prior to commencement (CIRB Ref: 2020/2533). Using our institution’s Sunrise Clinical Manager system (SCM), electronic medical records of patients who underwent unilateral hallux valgus correction between 2007 and 2013 in our tertiary institution were extracted. By reviewing the surgical chart of patients, patients who underwent either a scarf osteotomy or scarf and Akin osteotomy were identified and included in this study. Patients who did not meet the criteria for a diagnosis of adult hallux valgus or had additional conditions on top of hallux valgus were excluded from the study. These include patients who (1) were below the age of 18, (2) had HVA of less than 20°, and (3) had IMA of less than 10°, as summarized in Table 1. In addition, we included only patients with complete data points across a minimum 2 year follow up period. From an initial 311 cases, a total of 212 cases were identified and included in this study after applying our exclusion criteria. The mean age of our study group was 55.9 ± 12.8, and 92.9% were females. Patients were further stratified into 2 groups depending on the type of surgery they received, where 92 cases received only a scarf osteotomy.

**Table 1:** Inclusion and exclusion criteria for our study

Inclusion criteria	Exclusion criteria
Patients who underwent scarf or Akin osteotomy	Age < 18 years old
	HVA < 20°
Minimum 2 years follow up	IMA < 10°

*HVA* Hallux Valgus Angle, *IMA* Intermetatarsal Angle

### Radiographic parameters

**Fig. 1:**
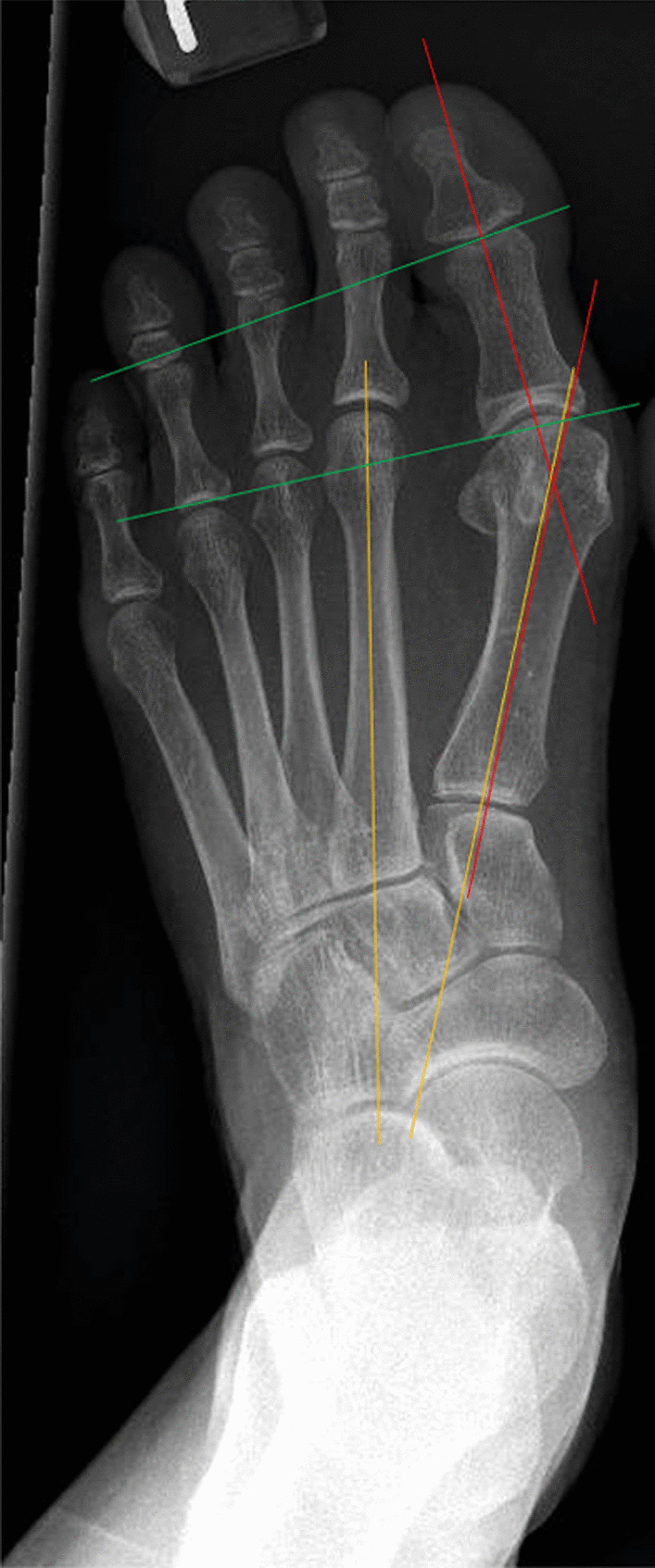
Radiological angles measured. Red = Hallux Valgus Angle (HVA). Yellow = Intermetatarsal Angle (IMA). Green = Proximal to Distal Phalangeal Articular Angle (PDPAA)

Pre-operative and post-operative radiological data were measured by a trained physician blinded to the patient's information on two separate occasions. Measurements were done on the Picture Archiving and Communication System (PACS: Carestream Health, Rochester, NY, USA). All pre-operative and post-operative radiographs were performed in the dorsal-plantar and lateral views. Radiological measurements collected included Hallux Valgus Angle (HVA), Intermetatarsal Angle (IMA), Proximal to Distal Phalangeal Articular Angle (PDPAA) and tibial sesamoid position as defined by Hardy and Clapham’s 7 position system [19]. Radiological parameters measured are as shown in Fig. 1. We further stratified our patients based on a pre-operative PDPAA of 8 degrees and carried out our statistical analysis to compare against the two surgical modalities carried out, within the PDPAA cut off as defined above.

### Clinical outcomes

A team of independent healthcare professionals consisting of orthopedic physiotherapists blinded to our study details and was not involved in the care of the patients assisted with collecting clinical outcomes from our patients. This involved administering a series of questionnaires containing patient reported outcome measures (PROM) which were done prospectively in person both pre-operatively and post-operatively. This was further assessed at regular intervals through clinical visits at 6 months and 2 years throughout the follow up period. These PROM consisted of Visual Analogue Scale (VAS), American Orthopedic Foot and Ankle Society (AOFAS) Hallux Metatarsophalangeal-Interphalangeal (MTIP-IP) score and Short Form 36-item health survey (SF-36). VAS was assessed from a scale of zero (totally no pain) to ten (worst pain possible). AOFAS MTP-IP consisted of 40 points for pain, 45 points for function and 15 points for alignment, a grand total of 100 points. SF-36 was divided into two higher order summary components, namely the Physical Component Score (PCS) and Mental Component Scale (MCS), each from a range of 0 (poor quality of life) to 100 (best quality of life).

### Operative procedure and care

All patients underwent unilateral hallux valgus surgery which were either scarf osteotomy or combined scarf and Akin osteotomy and additional soft tissue release was done as clinically indicated intra-operatively, and was similar across both groups. Clinical indication for surgery was for adult hallux valgus, without underlying metatarsophalangeal arthritis. Patients that underwent surgery for separate indications such as inflammatory arthritis were excluded. All patients underwent and failed a trial of conservative management which included footwear modification and analgesia, before being offered surgery. Post-operatively, the surgical site was dressed in compression dressing and weight bearing with a post-operative foot orthotic was allowed from post-operative day one.

### Statistical analysis

All continuous variables were expressed as a mean and standard deviation from the mean. Statistical significance was defined at an alpha of 0.05 (*p* value ≤ 0.05). Unpaired *T* Tests were conducted on normally distributed variables, while non-parametric tests such as Fisher's Exact Test, and Mann–Whitney U tests were performed on non-normally distributed variables. All statistical tests were performed using Python 3.9.7 and its publicly available statistical libraries (Pandas 1.4.3, SciPy 1.9.0 and NumPy 1.23.0).

## Results

### Patient demographics

A total of 71 patients had a PDPAA cut-off of 8° or more, where 29 received scarf and 42 received scarf and Akin osteotomy. Baseline demographic characteristics between the 2 groups were also not found to be significantly different and are summarized in Table 2.

**Table 2:** Patient demographics and procedures performed between respective procedures

Demographic factors	Scarf (N = 92)	Scarf + Akin (N = 120)	*p* value
Male (%)	5.43	8.33	0.59
Female (%)	94.6	91.7	0.59
Opside left (%)	56.5	54.2	0.781
Opside right (%)	43.5	45.8	0.781
Received Weil osteostomy (%)	51.1	41.7	0.211
BMI—mean (SD)	24.7 ± 3.6	24.3 ± 8.04	0.0653
Age—mean (SD)	56.9 ± 11.2	55.1 ± 13.8	0.527

*SD* standard deviation, *BMI* body mass index

### Radiological outcomes

Pre-operatively, at a PDPAA of less than 8 degrees, all radiological parameters were not statistically significant when comparing between the scarf versus the scarf and Akin group apart from PDPAA which was significantly higher in the scarf and Akin group (1.73 ± 4.78 vs 3.24 ± 3.76, *p* = 0.0324). These include tibial sesamoid position (6.11 ± 1.19 vs 6.01 ± 1.23, *p* = 0.634), HVA (33.4 ± 8.65 vs 34.6 ± 7.49, *p* = 0.392) and IMA (15.3 ± 2.63 vs 15.6 ± 2.86, *p* = 0.569) between the scarf and scarf and Akin group respectively. Post-operatively however, all radiological parameters were significantly better in the scarf and Akin group, namely tibial sesamoid position (3.57 ± 1.2 vs 2.95 ± 1.63, *p* < 0.0001), HVA (14.9 ± 8.95 vs 13.5 ± 13.4, *p* < 0.0001), IMA (8.59 ± 3.70 vs 7.08 ± 3.26, *p* = 0.00978) and PDPAA (3.97 ± 4.94 vs − 1.93 ± 5.91, *p* < 0.0001).

At a PDPAA of 8° and above, all radiological parameters were similar between both groups where tibial sesamoid position (6.24 ± 0.786 vs 6.14 ± 1.24, *p* = 0.707), HVA (32.3 ± 8.77 vs 34.7 ± 7.73, *p* = 0.169), IMA (15.2 ± 2.89 vs 16.1 ± 3.69, *p* = 0.337) and PDPAA (11.7 ± 2.845 vs 11.7 ± 2.92, *p* = 0.983) when comparing between scarf versus scarf and Akin pre-operatively, respectively. However post-operatively, only tibial sesamoid position and PDPAA showed significant improvement of (3.66 ± 1.29 vs 2.71 ± 1.57, *p* = 0.00371) and (7.92 ± 6.32 vs 0.757 ± 5.25, *p* < 0.0001) when comparing between scarf versus scarf and Akin respectively. Tables 3 and 4 reflects our radiological data.

**Table 3:** Comparison of radiological outcomes at PDPAA ≥ 8

	Scarf (N = 29)	Scarf + Akin (N = 42)	*p* value*
*Comparison preoperatively*			
Tibial sesamoid position—mean (SD)	6.24 ± 0.786	6.14 ± 1.24	0.707
HVA—mean (SD)	32.3 ± 8.77	34.7 ± 7.73	0.169
IMA—mean (SD)	15.2 ± 2.89	16.1 ± 3.69	0.337
PDPAA—mean (SD)	11.7 ± 2.85	11.7 ± 2.92	0.983
*Comparison postoperatively*			
Tibial sesamoid position—mean (SD)	3.66 ± 1.29	2.71 ± 1.57	**0.00371**
HVA—mean (SD)	16.0 ± 8.7	9.28 ± 13.0	0.472
IMA—mean (SD)	8.11 ± 2.89	7.48 ± 4.09	0.196
PDPAA—mean (SD)	7.92 ± 6.32	0.757 ± 5.25	**< 0.0001**

*HVA* Hallux Valgus Angle, *IMA* Intermetatarsal Angle, *PDPAA* Proximal to Distal Phalangeal Articular Angle, *SD* standard deviation

*Boldface indicate statistical significance

**Table 4:** Comparison of radiological outcomes at PDPAA < 8

	Scarf (N = 63)	Scarf + Akin (N = 78)	*p* value*
*Comparison preoperatively*			
Tibial sesamoid position—mean (SD)	6.11 ± 1.19	6.01 ± 1.23	0.634
HVA—mean (SD)	33.4 ± 8.65	34.6 ± 7.49	0.392
IMA—mean (SD)	15.3 ± 2.63	15.6 ± 2.86	0.569
PDPAA—mean (SD)	1.73 ± 4.78	3.24 ± 3.76	**0.0375**
*Comparison postoperatively*			
Tibial sesamoid position—mean (SD)	3.57 ± 1.2	2.95 ± 1.63	**< 0.0001**
HVA—mean (SD)	14.9 ± 8.95	13.5 ± 13.4	**< 0.0001**
IMA—mean (SD)	8.59 ± 3.7	7.08 ± 3.26	**9.78E−03**
PDPAA—mean (SD)	3.97 ± 4.94	-1.93 ± 5.91	**< 0.0001**

*HVA* Hallux Valgus Angle, *IMA* Intermetatarsal Angle, *PDPAA* Proximal to Distal Phalangeal Articular Angle, *SD* standard deviation

*Boldface indicate statistical significance

### Clinical outcomes

At a PDPAA of 8° and above, most pre-operative functional outcomes were not statistically significant between the scarf vs scarf and Akin group. These include VAS score (3.86 ± 2.96 vs 4.02 ± 2.65, *p* = 0.81), AOFAS (61.2 ± 16.2 vs 57.6 ± 13.2, *p* = 0.631) and MCS (53.9 ± 10.4 vs 55.7 ± 10.2, *p* = 0.452) respectively. Pre-operative PCS was the only functional outcome that had statistically significant differences (47.6 ± 8.74 vs 42.6 ± 10.5, *p* = 0.0451) respectively.

Most post-operative functional outcomes at 6 months did not yield statistical differences between the scarf versus scarf and Akin group. These included VAS (1.72 ± 2.49 vs 0.786 ± 1.69, *p* = 0.152), AOFAS (75.4 ± 17.3 vs 76.6 ± 18.1, *p* = 0.474), PCS (48.4 ± 11.4 vs 46.8 ± 11.1, *p* = 0.569), MCS (54.7 ± 11.6 vs 52.6 ± 11.7, *p* = 0.409), respectively. However, at 2 years post-operative follow up, AOFAS was significantly different (82.3 ± 15.3 vs 88.4 ± 13.0, *p* = 0.0224). VAS (1.07 ± 2.23 vs 0.476 ± 1.58, *p* = 0.179), PCS (51.0 ± 5.98 vs 46.7 ± 10.1, *p* = 0.138) and MCS (55.2 ± 9.5 vs 52.9 ± 8.98, *p* = 0.303) were not significant. Table 5 summarizes our data.

**Table 5:** Comparison of functional outcomes at PDPAA ≥ 8

	Scarf (N = 29)	Scarf + Akin (N = 42)	p-value*
*Comparison preoperatively*			
VAS	3.86 ± 2.96	4.02 ± 2.65	0.81
AOFAS	61.2 ± 16.2	57.6 ± 13.2	0.631
PCS	47.6 ± 8.74	42.6 ± 10.5	**0.0451**
MCS	53.9 ± 10.4	55.7 ± 10.2	0.452
*Comparison at 6 months*			
VAS	1.72 ± 2.49	0.786 ± 1.69	0.152
AOFAS	75.4 ± 17.3	76.6 ± 18.1	0.474
PCS	48.4 ± 11.4	46.8 ± 11.1	0.569
MCS	54.7 ± 11.6	52.6 ± 11.7	0.409
*Comparison at 2 years*			
VAS	1.07 ± 2.23	0.476 ± 1.58	0.179
AOFAS	82.3 ± 15.3	88.4 ± 13.0	**0.0224**
PCS	51.0 ± 5.98	46.7 ± 10.1	0.138
MCS	55.2 ± 9.5	52.9 ± 8.98	0.303

*PDPAA* = Proximal to Distal Phalangeal Articular Angle, *SD* standard deviation, *VAS* visual analogue scale, *AOFAFS* American Orthopedic Foot and Ankle Score, *PCS* Short Form-36 Physical Component Score, *MCS* Short Form-36 Mental Component Score

*Boldface indicate statistical significance

However, at a PDPAA of less than 8 degrees, patients who underwent scarf and Akin osteotomy had a significantly lower VAS score (1.16 ± 2.16 vs 0.321 ± 1.09, *p* = 0.00633), and higher AOFAS score (80.7 ± 14.3 vs 85.4 ± 12.5, *p* = 0.0123) compared to patients who only underwent scarf osteotomy, at 6 months respectively. In addition, patients who underwent additional Akin osteotomy had a significantly lower VAS score, and higher AOFAS score (83.0 ± 14.0 vs 90.7 ± 9.9, *p* < 0.0001) at 2 years respectively. This is shown in Table 6.

**Table 6:** Comparison of functional outcomes at PDPAA < 8

	Scarf (N = 63)	Scarf + Akin (N = 78)	*p* value*
*Comparison preoperatively*			
VAS	4.4 ± 2.98	4.26 ± 3.11	0.786
AOFAS	54.9 ± 14.8	59.1 ± 18.3	0.39
PCS	46.6 ± 8.81	46.0 ± 8.26	0.72
MCS	56.0 ± 8.91	53.7 ± 10.1	0.329
*Comparison at 6 months*			
VAS	1.16 ± 2.16	0.321 ± 1.09	**0.00633**
AOFAS	80.7 ± 14.3	85.4 ± 12.5	**0.0123**
PCS	52.4 ± 5.73	50.8 ± 8.41	0.865
MCS	54.1 ± 10.0	55.4 ± 9.87	0.338
*Comparison at 2 years*			
VAS	0.698 ± 1.73	0.333 ± 1.46	**0.0466**
AOFAS	83.0 ± 14.0	90.7 ± 9.9	**< 0.0001**
PCS	51.9 ± 5.38	50.5 ± 8.05	0.732
MCS	55.2 ± 10.6	55.8 ± 10.2	0.718

*PDPAA* Proximal to Distal Phalangeal Articular Angle, *SD* standard deviation, *VAS* visual analogue scale, *AOFAFS* American Orthopedic Foot and Ankle Score, *PCS* Short Form-36 Physical Component Score, *MCS* Short Form-36 Mental Component Score

*Boldface indicate statistical significance

## Discussion

Over the years, there have been a number of studies comparing outcomes of combined scarf and Akin osteotomy against scarf osteotomy [15, 20, 21], however criteria for carrying out the additional Akin osteotomy have been unclear [11, 22]. Various study methodologies include carrying out a mandatory Akin osteotomy for moderate to severe hallux valgus, or at the discretion of the surgeon based on intra-operative findings, which are not clear indications. A recent study by Kaufmann et al. aimed at determining when Akin osteotomy should be carried out based on radiological outcomes and risk of recurrence of the hallux valgus deformity in their study group [18, 23]. However, there are no studies that aimed to investigate this based on functional outcomes which arguably may be more important to patients, and equally clinically relevant. Our study provides value to existing literature in bridging this gap.

**Fig. 2:**
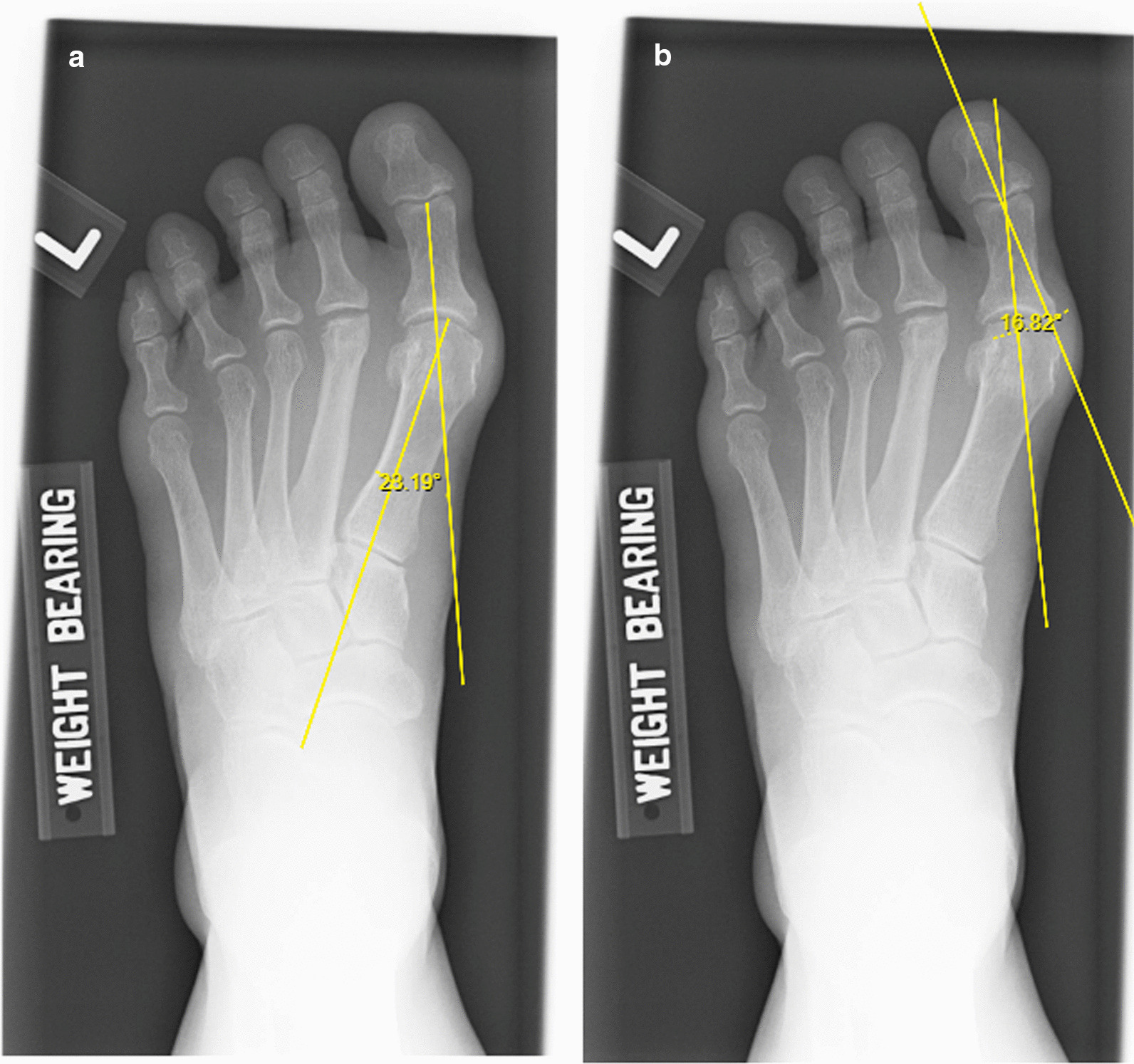
Radiograph demonstrating how isolated scarf osteotomy will not suffice given significant hallux valgus interphalangeus present. **a** Hallux Valgus Angle measured. **b** Hallux Valgus Interphalangeus Angle measured

**Fig. 3:**
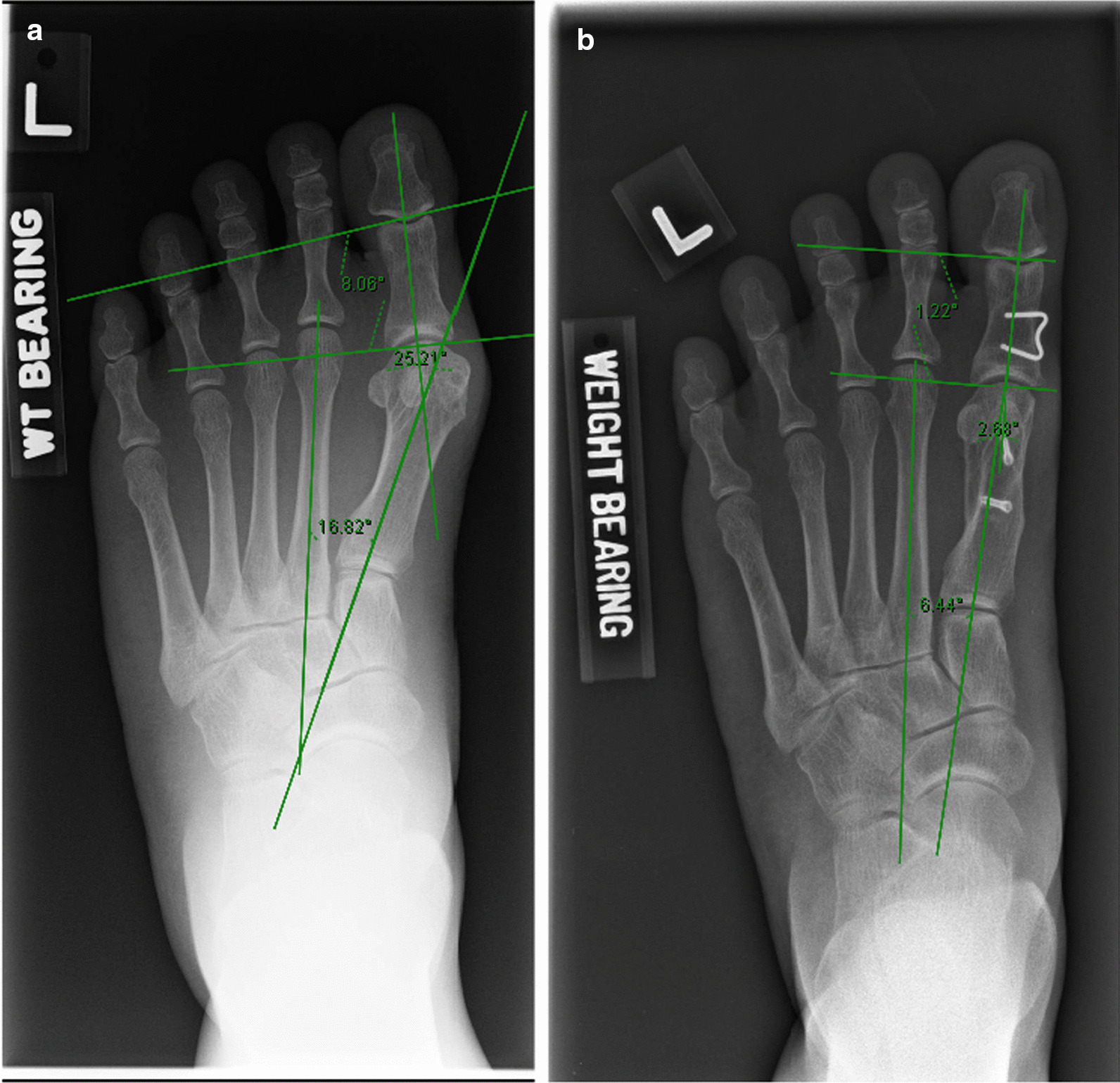
Radiograph demonstrating pre-operative and post-operative alignment after combined scarf and Akin osteotomy. **a** Pre-operative radiograph. **b** Post-operative radiograph

While there are numerous techniques described to surgically correct a hallux valgus deformity [5–8], an Akin osteotomy is described to be an osteotomy affecting the phalanges [14], while the scarf osteotomy corrects the deformity at the metatarsus [5, 7]. Theoretically, indications to carry out an Akin osteotomy would logically be based on the degree of phalangeal pathology contributing to the hallux valgus deformity, as demonstrated in Fig. 2. Figure 3 demonsrates pre and post-operative alignment post scarf and Akin osteotomy. The hallux valgus interphalangeus angle has been frequently used to assess the degree of phalangeal pathology, however a study carried out by Castillo-Lopez et al. [12] revealed that measuring hallux valgus interphalangeus angle had significant variations across patients including those without hallux valgus deformities, due to the deviation of the distal phalanx against the proximal phalanx which is seen in normal patients too. In a separate study carried out by Kaufmann et al. [13], PDPAA was found to be a better marker representing the pathology. However, the extent of hallux valgus deformity contributed by a poor PDPAA where additional Akin osteotomy would be indicated is still lacking.

The most important finding in our study was that when comparing between patients with a PDPAA of 8° or more, who underwent a scarf and Akin osteotomy vs scarf osteotomy, there was no difference between improvement in functional outcomes immediately post-operatively. However, at 2 years, which would represent a mid to long term follow up period, patients who underwent a scarf and Akin osteotomy experienced a better AOFAS score as compared to patients who only underwent a scarf osteotomy. Despite other functional outcomes not showing significant difference between the scarf and Akin osteotomy and the scarf osteotomy group, results reflected for AOFAS cannot be discounted as it is one of the most widely used functional outcome measures [24]. We postulate that this is likely due to the additional soft tissue rebalancing that patients that have undergone scarf and Akin osteotomy benefit from, which can likely only be achieved with a correction of the hallux valgus interphalangeus seen intra-operatively [11, 25]. This is especially so with rehabilitation and physiotherapy post-operatively which takes time for optimal surgical outcomes to be achieved.

Another finding in the existing study shows that pre-operative PCS was significantly lower in the combined scarf and Akin group compared to the scarf only group. The use of PCS and MCS as an aggregate under the original SF-36 score was previously described by Ware and Kosinski [26], and the use of PCS has been shown to be more reliable over the original MCS, as well as for use in lower limb conditions [27]. We postulate that the reason why patients that have underwent combined scarf and Akin osteotomy having a significantly lower PCS over patients who underwent only scarf osteotomy is likely due to these patients having more severe hallux valgus deformity, hence causing more functional limitations physically and warranting an additional Akin osteotomy. Post-operatively, both groups of patients likely would have experienced relief from such symptoms with surgical correction of their foot pathology. As such, pre-operative PCS or degree of functional limitation physically could also serve as a guide as a surgical indication for Akin’s osteotomy.

In our study, there was significant correction of the various radiological indicators of hallux valgus across both the scarf only and combined scarf and Akin group. This was a finding that is echoed in existing literature where a combined scarf and Akin procedure has led to more significant improvements in the hallux valgus deformity especially after correcting for a phalangeal deformity which a conventional scarf osteotomy would not be able to target [15, 20]. Patients can experience debilitating symptoms from both a hallux valgus as well as hallux valgus interphalangeus deformities and a scarf osteotomy would only correct the malalignment from the first metatarsal and proximal phalanx, whilst leaving the valgus deformity of the hallux untouched, if present pre-operatively.

We were surprised that with a PDPAA of less than 8°, there were more functional and radiological outcomes that were significantly different between the patients that received scarf and Akin osteotomy and scarf osteotomy alone. This is because one may expect patients with a worse hallux valgus deformity to benefit from an additional Akin osteotomy. The current study has revealed that for a PDPAA of less than 8°, patients that received additional Akin osteotomy experienced significantly better pain control post-operatively and AOFAS functional scores both in the short and mid-long run. In addition, radiological parameters for a hallux valgus deformity reflected more significant improvements when additional Akin osteotomy was carried out for a PDPAA of less than 8°. In the study carried out by Kaufmann et al. [23], a PDPAA threshold of 8 degrees was determined by taking into account the expected PDPAA improvement from a metatarsal osteotomy while accepting recommendations from a separate study studying hallux valgus interphalangeus [28]. However, exploring lower PDPAA thresholds against radiological outcomes was not reported. Our results reflect that the PDPAA threshold to warrant an additional Akin osteotomy may actually be lower, and patients may still be able to achieve good functional and radiological outcomes post-operatively. However, correlating our functional outcomes with the radiological findings found in the previous study, we would still recommend a PDPAA of 8 degrees, to achieve both optimal functional and radiological outcomes. Future studies with larger sample sizes could also be done to further validate this or to explore a PDPAA cut off of less than 8 degrees. With a lower PDPAA threshold, it may benefit more patients in allowing them access to an additional Akin’s osteotomy, with the better hallux valgus correction that it brings, whilst balancing the potential risks of complications from carrying out the additional procedure [29, 30].

We recognize the limitations of our study. Firstly, our study is one of retrospective nature which has inherent biases when reviewing our data. Secondly, our follow up period is up to 24 months, and though it is meant to represent longer term outcomes, is still a relatively short follow up period. In addition, as compared to larger observational studies performed whilst looking into national registries, our study is carried out in a single institution by accredited foot and ankle surgeons. While this improves the internal validity of our study, our results are limited in terms of external validity especially in terms of application to a western context, as our patient groups are Asian. However, we have limited this by showing that our pre-operative demographic factors are not statistically significant across both groups, and we have implemented standard post-operative protocols for rehabilitation, hence minimizing any confounding effect. Lastly, despite having collected functional outcome measures across the 24 months follow up period, radiological data across this period was lacking. Ideally, we would like to have collected these radiological data to monitor alignment and compare it against functional outcomes across the follow up period. However, this was not the main aim of our study, though we concede having this data may add value to our study.

## Conclusion

In conclusion, combined scarf and Akin osteotomy remains an attractive option for patients with hallux valgus deformity and should be carried out for patients with a PDPAA of 8° or more. Patients with limited physical function or poor SF-36 PCS score should also be considered for it. Future studies of larger sample sizes should further investigate whether a PDPAA of a lower threshold can provide good radiological and functional outcomes.
